# Leveraging Perspective Transformation for Enhanced Pothole Detection in Autonomous Vehicles

**DOI:** 10.3390/jimaging10090227

**Published:** 2024-09-14

**Authors:** Abdalmalek Abu-raddaha, Zaid A. El-Shair, Samir Rawashdeh

**Affiliations:** Department of Electrical and Computer Engineering, University of Michigan-Dearborn, Dearborn, MI 48128, USA; zelshair@umich.edu (Z.A.E.-S.); srawa@umich.edu (S.R.)

**Keywords:** autonomous vehicles, perspective transformation, deep learning, pothole detection, computer vision, mobile robotics

## Abstract

Road conditions, often degraded by insufficient maintenance or adverse weather, significantly contribute to accidents, exacerbated by the limited human reaction time to sudden hazards like potholes. Early detection of distant potholes is crucial for timely corrective actions, such as reducing speed or avoiding obstacles, to mitigate vehicle damage and accidents. This paper introduces a novel approach that utilizes perspective transformation to enhance pothole detection at different distances, focusing particularly on distant potholes. Perspective transformation improves the visibility and clarity of potholes by virtually bringing them closer and enlarging their features, which is particularly beneficial given the fixed-size input requirement of object detection networks, typically significantly smaller than the raw image resolutions captured by cameras. Our method automatically identifies the region of interest (ROI)—the road area—and calculates the corner points to generate a perspective transformation matrix. This matrix is applied to all images and corresponding bounding box labels, enhancing the representation of potholes in the dataset. This approach significantly boosts detection performance when used with YOLOv5-small, achieving a 43% improvement in the average precision (AP) metric at intersection-over-union thresholds of 0.5 to 0.95 for single class evaluation, and notable improvements of 34%, 63%, and 194% for near, medium, and far potholes, respectively, after categorizing them based on their distance. To the best of our knowledge, this work is the first to employ perspective transformation specifically for enhancing the detection of distant potholes.

## 1. Introduction

### 1.1. Pothole Detection Importance for Autonomous Vehicles

Potholes, commonly found in asphalt pavements, are caused by water weakening the underlying soil and repeated traffic wear. Factors such as temperature fluctuations causing expansion and contraction, poor drainage allowing water infiltration, and the use of low-quality materials further contribute to the formation of depressions or holes in the road surface [1,2,3]. These can vary in severity and pose significant hazards, such as suspension damage, tire punctures, and even accidents, by causing loss of control or immobilization of vehicles. The dangers of potholes extend to both vehicles and pedestrians, highlighting the critical need for efficient detection systems.

In 2011, poor road conditions caused around 2200 deaths in India, while in the U.S., one-third of the 38,824 traffic deaths in 2020 were linked to substandard roads. Michigan, with some of the worst potholes, spent millions annually on repairs, highlighting the widespread impact of this issue. Effective pothole detection is crucial, particularly for autonomous vehicles, which rely on accurate hazard detection to ensure safe operation. This need is underscored by the potential damages and safety risks associated with potholes, emphasizing the importance of accurate and timely detection systems to mitigate these dangers.

### 1.2. Human Response to Potholes

When a driver encounters a pothole, the human reaction time to apply the brakes can vary. For simple tasks, the average human reaction time is often quoted as 0.2 s [4]. However, for more complex tasks, such as emergency braking when a pothole is detected, the reaction time is typically longer. These times can be greatly affected by the driver’s alertness and the expectation of the need to brake [5]. Hence, detecting potholes from a greater distance is crucial, as it provides the driver more time to react and navigate safely around the hazard. In addition, far pothole detection allows drivers to prepare and adjust their driving accordingly, reducing the likelihood of sudden maneuvers that can lead to accidents.

In contrast to human drivers, an AV equipped with pothole detection systems can identify and respond to potholes in real time. These systems can modulate vehicle speed and position upon detecting a potential pothole and sometimes ensure the vehicle remains within its lane while minimizing impact. This rapid and precise response can significantly improve road safety by allowing the vehicle to take immediate action to avoid the pothole or minimize impact, thus reducing potential damage and improving overall vehicle control.

### 1.3. Pothole Detection Methods

The implementation of pothole detection systems in AV offers several advantages. Not only does it improve road safety by reducing the risk of accidents caused by potholes, but it can also contribute to more efficient road maintenance by providing accurate and timely data on the locations and severity of potholes. Moreover, detecting and avoiding potholes can reduce the fuel consumption, wear and tear, and maintenance costs of a vehicle [6]. In addition, it can indirectly decrease the total travel time in some cases [6].

Potholes are detected and observed in different ways, including manual human detection, vibration-based detection, sensor-based detection, and vision-based detection. Human observation is the traditional method for detecting potholes. [7]. Drivers must look out for potholes while driving and react quickly to avoid them, which can lead to dangerous situations. This approach is inconsistent due to human error and is inefficient for detecting multiple objects. In addition, vibration-based detection methods use accelerometers to detect potholes based on the vibration information of the acceleration sensors attached to the vehicle [8]. This method is cost-effective and suitable for real-time processing. However, it has limitations in providing the exact shape of potholes and could provide incorrect results, as road joints can be misidentified as potholes [9]. Moreover, this method is not suitable for detecting potholes in order to avoid or act towards reducing their effect on the vehicle. Sensor-based detection methods, such as light detection and ranging (LiDAR) and radio detection and ranging (RADAR), use electromagnetic waves to detect potholes. LiDAR uses light waves [10], providing high-resolution data and precision [11]. However, it can be expensive, making it less feasible for widespread use in AVs. RADAR, on the other hand, uses radio waves and is superior in terms of cost and ability to monitor large areas [11]. Nevertheless, its lower resolution compared to LiDAR makes it difficult to track and distinguish objects in crowded environments, a common scenario for AVs [12]. Additionally, computer vision techniques for pothole detection have gained popularity due to the accessibility and feasibility of cameras, especially for AVs. These techniques use images or videos as input data and apply deep learning and image processing techniques to detect potholes. Although each method has its strengths and weaknesses, computer vision techniques offer significant advantages in terms of cost-effectiveness, precision, and the ability to integrate with other data sources for pothole detection. Hence, our work is purely focused on vision-based detection techniques.

### 1.4. Challenges in Potholes Detection

Deep learning and vision-based approaches for pothole detection face significant challenges, particularly related to the handling of object detection tasks involving small and distant objects. A critical limitation stems from the requirement to resize images to a fixed, smaller size to ensure reasonable processing times. This resizing is necessary because models trained on high-resolution images demand substantial computational resources, leading to impracticalities in real-time applications. As a result, small objects, such as potholes, especially those far away, often become indistinguishable when images are downscaled. The reduction in size leads to a loss of crucial details, making it challenging for the model to accurately detect and classify these objects. This issue is exacerbated by the fact that more complex models, while potentially offering increased accuracy, do not necessarily resolve the problem of lost detail due to image downscaling.

Moreover, the utilization of high-resolution images in training object detection models is hindered by the immense complexity of the search space. High-dimensional data require more extensive computational resources and can significantly slow down the training and inference processes. This trade-off between image resolution and processing efficiency is particularly problematic in real-time applications such as autonomous driving, where rapid detection and response are crucial for safety. The need to rescale high-definition images to lower resolutions introduces a bottleneck in object detection systems. The act of rescaling can lead to a substantial loss of fine-grained features that are essential for accurately identifying potholes, thereby compromising the model’s performance. One proposed solution to mitigate these challenges is the use of perspective transformation. Unlike conventional resizing, perspective transformation selectively focuses on a region of interest (ROI) within an image, such as the area containing a pothole. This approach preserves critical features by altering the viewing angle, effectively enlarging the ROI and reducing the prominence of irrelevant areas. While this method does not introduce new features, it helps retain more of the significant details associated with the potholes, thereby improving detection accuracy. Although theoretically training a model on full-resolution images would be ideal, it is practically unfeasible due to computational constraints. Hence, perspective transformation offers a practical compromise, allowing the retention of essential features while maintaining manageable processing times, thus enhancing the robustness and effectiveness of pothole detection in autonomous vehicle systems.

### 1.5. Proposed Method: Vision-Based Pothole Detection Using Perspective Transformation

To address these challenges, we present a novel approach that leverages perspective transformation to enhance pothole features. Perspective transformation is an image processing technique that alters the viewing angle of potholes from the perspective of the vehicle or driver to a bird’s-eye view [13]. This transformation is based on four source points extracted from the values of the bounding box and the location of the pothole pixels. These points represent the ROI to be transformed. Our method proposes an automated selection of these four points by considering all bounding box values in the dataset, thereby reducing human intervention and creating an ROI that is optimal for all images and their bounding boxes.

**Figure 1:**
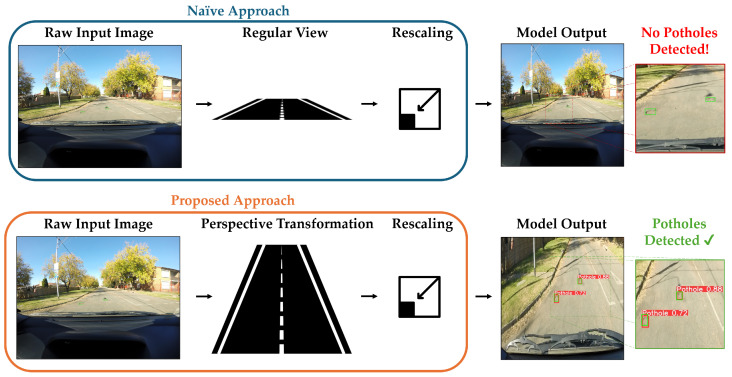
Comparison between the naive approach and our proposed approach. The naive approach involves loading the raw input image and then simply downscaling it to the required input resolution for the object detection network, losing significant image features and resulting in undetected potholes. Meanwhile, our approach demonstrates successful and robust pothole detection by transforming the input image to primarily retain the region of interest and minimize irrelevant segments of the image. Ground-truth pothole labels and predicted potholes are represented by green and red bounding boxes, respectively. Street and rescaling icons created by Trevor Dsouza and Doodle Icons via TheNounProject.com (accessed on 1 August 2024).

The perspective transformation modifies the image, enlarging the ROI and minimizing the scale of irrelevant portions. This technique effectively simulates bringing the potholes closer, thereby improving feature extraction. By applying perspective transformation, we generate a magnified and standardized view of the pothole region within the image. This enhanced view enables the computer vision algorithm to more effectively extract image features such as color, edges, contours, and size, significantly improving the accuracy of pothole detection, particularly for those located at a distance. Figure 1 shows the framework overview of our approach compared to the naive approach.

We summarize the contributions of our work as follows:

1. We developed an automated perspective transformation algorithm that selects the ROI—the street area containing potholes from the vehicle’s perspective—resulting in the generation of a transformation matrix. The matrix warps the image, excluding irrelevant areas like the sky, while enhancing pothole features by making distant ones appear closer for easier detection.
2. Using this matrix to transform the input data before model inference significantly boosts the accuracy and robustness of pothole detection with limited impact on runtime. To the best of our knowledge, this is the first study to utilize perspective transformation in this manner to enhance pothole detection performance.
3. We propose an intuitive evaluation strategy to assess the pothole detection models’ performance across potholes at three distance ranges (near, medium, and far), demonstrating the potential of our approach in improving pothole detection at far distances.

## 2. Related Work

Numerous studies have investigated various computer vision and image processing techniques for automated pothole detection using visual road imagery. However, the majority of these approaches have focused more on pothole classification rather than detection. Furthermore, most of the papers have not exclusively used the same dataset as our study, making direct comparisons between our work and these studies challenging. Additionally, other research has highlighted the significance of utilizing perspective transformation in object detection applications. The following sections review some of the aforementioned approaches.

### 2.1. Vision Approaches

Basic vision approaches in pothole detection have leveraged simple image-processing techniques to identify road defects. For example, Nienaber et al. [14] utilized road color modeling combined with edge detection, while Pereira et al. [15] employed a basic four-layer CNN while using images captured by a mobile phone, limiting the practicality of the work. These methods provide cost-effective solutions without the need for complex sensors or deep learning architectures. However, they suffer from limitations in generalizability across diverse road conditions. Furthermore, the precision needed for real-time application is lacking and the absence of comprehensive visual analyses and separate test sets undermines the reliability of their results, which could be affected by the lack of a pothole dataset from a vehicle perspective.

Deep learning techniques have shown great potential in developing more powerful models for pothole detection and classification. Chen et al. [16] focused on classification tasks, using location-aware CNNs to identify areas likely to contain potholes. Additionally, Dhiman et al. [17] reported promising results by employing transfer learning with Mask R-CNN and YOLOv2. Although these models demonstrate high classification accuracy, they are computationally expensive and struggle with precise localization. Additionally, the two-stage nature of some pipelines, such as those of Chen et al., can introduce further computational overhead. The performance of these models is also heavily dependent on high-quality input data, and challenges in real-time applications due to processing latency remain a significant drawback.

Stereo vision approaches, explored by Dhiman et al. [17,18], utilize depth information to identify road defects by analyzing road elevation and depth variations. Although these methods theoretically provide detailed spatial information, they are highly dependent on the quality of stereo images and the precise camera calibration. Issues such as noise, distortion, and the need for well-aligned image pairs can significantly affect the accuracy of depth estimation and pothole detection. The computational intensity further hinders the practical deployment of these techniques in real-time applications, particularly in the context of autonomous vehicles.

Data augmentation and enhancement techniques have also been explored to improve pothole detection performance. Maeda et al. [19] utilized Generative Adversarial Networks (GANs) to generate synthetic training data in combination with a Single-Shot Multibox Detection (SSD) model. The addition of synthetic data led to moderate improvements, increasing the F-score by 5% when the synthetic data were less than 50% of the original dataset and by 2% when they constituted about 50%. However, performance deteriorated when the synthetic data exceeded 50% of the original data. Despite these improvements, the approach faced challenges, including increased computational complexity, instability during GAN training, and concerns about the generalizability of the model to real-world conditions. Similarly, Salaudeen et al. [20] introduced a pothole detection approach that combines an image enhancement GAN with an object detection network. The GAN, specifically ESRGAN, enhances image quality to make potholes more distinguishable, while the detection network identifies and localizes them. Using a combination of datasets, their method produced notable results. EfficientDet demonstrated an improvement in mAP when applied to super-resolution images compared to low-resolution ones. Similarly, YOLOv5, in conjunction with ESRGAN, showed better performance in super-resolution images compared to low-resolution counterparts, both evaluated within the same IoU range. Despite improved detection metrics, this approach introduces significant computational overhead and risks of overfitting, particularly since results depend mainly on the quality of the generated data.

Specific YOLO variants have also been investigated. Al-Shaghouri et al. [21] investigated real-time pothole detection using YOLOv3 and YOLOv4. However, the evaluation was performed at a low IoU threshold of 25%, which is less stringent than typical object detection standards. This low threshold, along with performance variability at different distances, highlights some limitations of their approach despite the promising precision results. Buko et al. [22] examined the effectiveness of YOLOv3 and Sparse R-CNN under various challenging conditions, revealing a substantial performance degradation under low light and adverse weather conditions, indicating limited applicability in various real-world scenarios. Nevertheless, this project used the same dataset for training and testing, which affects the generalizability of this approach. Rastogi et al. [23] modified YOLOv2 to address issues such as vanishing gradients and irrelevant feature learning. However, the reliance on close-range smartphone images limits the model’s applicability to broader contexts, such as autonomous vehicles where variable distances and angles are encountered.

While previous research in pothole detection has largely concentrated on improving algorithmic architectures or combining multiple techniques to enhance detection accuracy, our contribution addresses a fundamental gap by focusing on the quality and effectiveness of the input dataset. By leveraging perspective transformation, our approach optimizes the dataset, maintains the desired objects’ features, and enhances them. This method effectively tackles the common issue of limited data without incurring additional computational costs during training. In fact, it often reduces training time compared to using regular or cropped images, making it a practical and efficient solution. Unlike other approaches that are computationally intensive and dependent on high-quality data, our contribution ensures better utilization of the current dataset, enhancing the robustness and real-world applicability of pothole detection systems without the trade-offs associated with complex algorithmic fusions.

### 2.2. Perspective Transformation Technique

Perspective transformation is a common image processing technique that adjusts an image’s viewpoint, often enhancing object detection accuracy and data augmentation. For instance, in [24], the authors presented a method to construct perspective transformations for detecting 3D bounding boxes of vehicles in traffic surveillance, enhancing the accuracy of object detection by extending traditional 2D detectors. Lee et al. [25] introduced a multi-view approach that leverages perspective transformation for pedestrian detection, projecting features onto a ground plane to improve localization accuracy. Additionally, Wang et al. [26] utilized perspective transformation in data augmentation, enhancing object detection by simulating variations in object size and viewpoint. In another study, Hou et al. [27] proposed a feature transformation method for multiview aggregation in 3D object detection, focusing on head–foot pair detection. These studies highlight the significance of perspective transformations in addressing challenges related to varying viewpoints in object detection. However, our work goes a step further by directly enhancing object detection quality through an automated application of perspective transformation, improving object feature representation for superior model training and performance.

## 3. Methodology

Potholes significantly impact vehicles and road users, increasing the likelihood of hazardous situations. Therefore, implementing an effective early detection system for potholes is crucial to mitigate potential risks and prevent undesirable or harmful incidents. In this section, we present a detailed breakdown of our proposed approach detailing the techniques employed in this work.

### 3.1. Framework Overview

**Figure 2:**
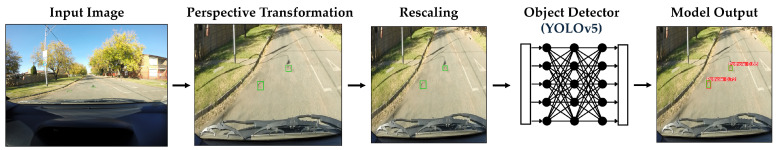
Overview of the proposed framework. Raw input images are initially transformed using the transformation matrix generated by our proposed automated algorithm. Then, the resulting images are rescaled to the required input resolution and fed to the object detection network (e.g., YOLOv5). Ground truth pothole labels and predicted potholes are represented by the green and red bounding boxes, respectively. Neural network icon by Lucas Rathgeb via TheNounProject.com (accessed on 1 August 2024).

When utilizing object detection models, the typical approach is to feed the captured raw images directly once they are rescaled to the desired size. This usually causes a significant loss of image features that are critical for enabling robust pothole detection, especially when using high-resolution input images. Using this naive approach, detecting far potholes, for instance, would be a very challenging task. Instead, our approach, demonstrated in Figure 2, enhances pothole detection from a vehicle’s perspective by making potholes appear closer, larger, and with amplified features. Initially, we apply a perspective transformation technique to convert input images from the vehicle’s view to a view closer to a bird’s-eye view, making them virtually closer and larger. Then, these transformed images are then fed to YOLOv5 [28], a well-known object detector, which uses those images to train and improve the model’s ability to detect potholes, especially those at a distance, compared to using regular images.

#### 3.1.1. Perspective Transformation Motivation

**Figure 3:**
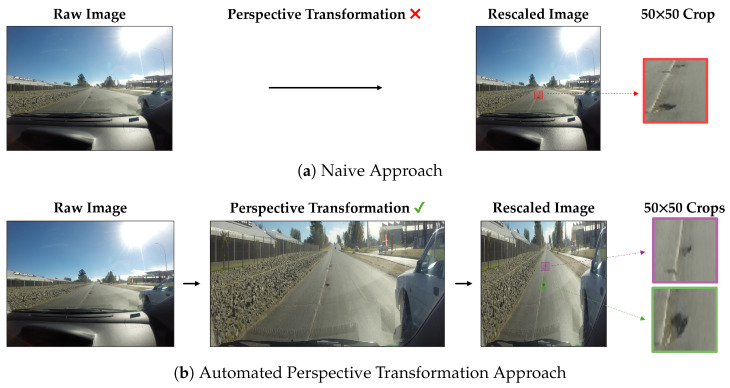
Comparison of the resulting preprocessed input images between (**a**) the naive approach and (**b**) the automated perspective transformation approach. The naive approach involves reading the image as is and then downscaling to a fixed input resolution (800×800 in this example). A 50×50 image crop demonstrates very low resolution for the potholes in the scene. Instead, our proposed approach transforms the image to mainly focus on the ROI (i.e., the street) where, after rescaling to the same input resolution, the resulting spatial resolutions of the potholes are much larger with clearer image features as depicted by the 50 × 50 image crops.

Raw images captured by cameras are typically of a high resolution, providing a great level of detail. However, due to design limitations and the need for real-time processing, most object detection networks are trained on fixed, low-resolution images [28,29,30]. For example, optimized real-time variants of SSD [29] and YOLOv3 [30] are designed for 300×300 and 320×320 resolutions, respectively. Meanwhile, the more recent YOLOv5 [28] has been tailored for an input spatial resolution of 640×640. Although the trend demonstrates an increase in the models’ input resolutions, they are still quite small relative to raw camera output resolutions. Further, training these models on larger input resolution negatively impacts inference times [28,29], which is not ideal for real-time applications. This necessitates resizing the images to a predetermined, smaller size for effective training and generalization. However, when these images are resized, the features of small objects, such as potholes, can become significantly less discernible. Potholes may appear very small relative to the overall image size, resulting in insufficient features for the model to detect and differentiate them effectively, as shown in Figure 3. Furthermore, portions of the image that do not contain regions of interest, such as sidewalks or the sky, are often retained, limiting the focus on the relevant areas necessary for pothole detection. To improve detection accuracy, it is beneficial to adjust the image perspective to emphasize the road—the primary ROI—while minimizing the inclusion of non-essential parts of the image. This approach ensures that more useful features are preserved after rescaling, enhancing the model’s ability to detect potholes without significantly increasing computational overhead [31,32].

#### 3.1.2. YOLOv5: Key Features and Functionality

In the field of computer vision, deep learning has become the preferred approach for object detection, particularly for complex and variable tasks like pothole detection. Traditional handcrafted image processing methods often struggle with the variability in pothole shapes and sizes and the diverse lighting conditions found in real-world environments. Deep learning-based detectors, such as the YOLO series, provide a more robust and dynamic solution that can generalize well across different scenarios. Among these, YOLOv5 stands out for its balance of speed and accuracy, making it ideal for applications requiring real-time performance. This study employs YOLOv5, implemented in PyTorch [33], for its efficiency, speed, and relatively low computational demands, with versions available in nano, small, medium, large, and extra-large configurations [28].

The architecture of YOLOv5 follows a similar structure to previous YOLO versions, with a backbone network and detection heads. The backbone, based on the efficient CSPDarknet53 architecture, extracts features from the input image using 53 convolutional layers. The detection heads then predict bounding boxes and class probabilities. YOLOv5 employs a modified YOLO head, consisting of convolutional layers that vary depending on the model configuration (e.g., YOLOv5-small, YOLOv5-medium, YOLOv5-large, YOLOv5-extra-large). Unlike earlier methods, YOLOv5 performs object detection in a single forward pass, ensuring high speed and efficiency which are crucial for real-time applications like surveillance and autonomous driving. With different variants representing varying model scales and complexities, YOLOv5 offers options like YOLOv5-small, which has 7.2 M parameters and 16.5 GFLOPs, and YOLOv5-large, with 46.5 M parameters and 109.1 GFLOPs. Notably, YOLOv5-small performs better when applied to our approach compared to larger models with a naive approach, highlighting that a more complicated model is not always necessary for good results. We focus on using YOLOv5-small for its balanced real-time performance and accuracy, while also evaluating other YOLOv5 variants to assess the impact of our methods on their performance.

YOLOv5 offers several key advantages, including enhanced speed and accuracy due to its lightweight architecture and efficient training through focus modules and data augmentation. Its flexibility and usability are boosted by its user-friendly and customizable PyTorch framework, along with a modular design that allows easy customization for various hardware platforms. Additional benefits include improved anchor box prediction for more accurate bounding box localization and strong community support for collaboration, quick bug fixes, and feature development. Although newer versions such as YOLOv7 [34] and YOLOv8 [35] offer advancements, the speed, efficiency, and strong community support of YOLOv5 make it the preferred choice for this study. While this work is applicable to other detection networks, we chose YOLOv5 for these reasons.

### 3.2. Automated Perspective Transformation Algorithm

Perspective transformation is a fundamental tool in computer vision that allows for the alteration of an image’s viewpoint, simulating a change in the observer’s position. This is performed using a 3 × 3 transformation matrix that maps points from the original image plane to a new plane. The matrix is calculated by identifying four points in the original image and their corresponding locations in the transformed image. By solving a set of linear equations, the matrix adjusts the coordinates of these points, allowing the image to appear as though it is viewed from a different angle. This method preserves the straightness of lines and their intersections, making it useful for applications like image rectification [36], object tracking [37], and traffic surveillance [38].

For pothole detection, this technique concentrates on the region of interest (ROI), specifically the street, while reducing the prominence of irrelevant areas such as the roadside and sky. By employing perspective transformation, objects that are distant are virtually brought closer, thereby making potholes appear significantly larger in the processed image. This enhancement is accomplished by detecting and matching features, estimating homography, and warping the image using a 3×3 homography matrix *M*. This process emphasizes relevant features, thereby enhancing detection accuracy, as follows:

(1) $$t^{\prime }=M\cdot t\;,$$

where $t^{\prime }$ and *t* are the coordinates of the ROI in the transformed and source images, respectively. The homography matrix *M* is calculated using the corresponding points as explained earlier, aiding in feature extraction and pothole bounding-box regression by mimicking a bird’s eye view for more robust pothole detection in AVs. Moreover, it holds the transformation parameters that map points from the source image to the target image [39].

The homography matrix *M* can be represented as follows:

(2) $$M=\left(\begin{array}{ccc} m_{11} & m_{12} & m_{13}\\ m_{21} & m_{22} & m_{23}\\ m_{31} & m_{32} & m_{33} \end{array}\right)$$

Each element ($m_{ij}$) within the matrix contributes to the transformation:

- $m_{11},m_{12},m_{13}$: Affect the x-coordinate of the transformed point.
- $m_{21},m_{22},m_{23}$: Affect the y-coordinate of the transformed point.
- $m_{31},m_{32},m_{33}$: Homogenization factors (usually, $m_{33}$ is set to 1).

Applying perspective transformation to a set of images requires manually selecting the boundary points of the ROI. In our application, this ROI would primarily be the road as viewed from the perspective of the vehicle itself. However, the boundaries of the road change significantly from one scene to another depending on many factors, such as the type of road, curvature, number of lanes, etc. Therefore, an ideal application of this technique would be to select the corners of the road for each given image. However, this approach is not feasible for object detection applications due to its time-consuming and error-prone nature, especially when dealing with different scenes that include not only straight streets but street curvatures, u-turns, etc. Manually specifying four points for each image based on the shape of the road is labor-intensive and introduces significant variability and inaccuracies, making it unsuitable for large-scale datasets. Furthermore, due to its infeasibility, a new transformation matrix is required to be generated for each image, which would add run-time overhead. Therefore, automating this process is essential to ensure a usable workflow and to generalize the technique to most, if not all, datasets with similar structures. To address this challenge, we propose an algorithm that automatically finds a set of ROI corner points that enables the generation of the perspective transformation matrix *M*. Using this matrix, a set of images from the same source can be similarly transformed to better represent the ROI. Consequently, this approach ensures consistency and precise ROI selection in any given dataset of the same source.

To achieve an optimal transformation, it is crucial to accurately identify the best ROI that encompasses all bounding boxes of potholes within an image, ensuring that no potholes are excluded. The automatic transformation process involves determining the ROI by calculating the coordinates of all bounding boxes to establish four defining points. Specifically, the boundary coordinates of the ROI are determined as follows: the top-left point is defined by the minimum *x* and *y* coordinates among all bounding boxes, the top-right point by the maximum *x* and minimum *y* coordinates, the bottom-left point by the minimum *x* and maximum *y* coordinates, and the bottom-right point by the maximum *x* and *y* coordinates. An offset value α is added to each of these points to ensure that the ROI extends slightly beyond the boundaries of the bounding boxes. This offset allows for full coverage of the image or ROI boundaries, depending on how much extension the user desires. This approach guarantees that the ROI fully covers the outermost boundaries of the bounding boxes, thereby ensuring comprehensive inclusion of all potholes. Additionally, the alpha value was tested at 0.2, 0.1, and 0 to control the inclusion of background features, particularly near the top of the image. A higher alpha value (e.g., 0.2) allows more background context, helping the model better differentiate between objects and background, especially when bounding boxes are close to the image edge or not perfectly accurate. The optimal alpha value was chosen to balance the inclusion of background features with the model’s ability to handle imperfect bounding box annotations.

To implement automatic perspective transformation, it is expected that we have a labeled object detection dataset. The algorithm could then be applied to the selected images and their corresponding labels, following the subsequent steps outlined in the algorithm structure below Algorithm 1. The transformed images and label files were then saved to their designated output directories.
**Algorithm 1** Automatic perspective transformation for images and bounding boxes.**Input:** Images, ground truth labels, ROI offset α**Output:** Transformed images and bounding boxes, perspective transformation matrix *M*
**Initialize lists** 1: Initialize lists: all_x_min, all_y_min, all_x_max, all_y_max, all_w, all_h     ▹x_min, y_min: top-left corner coordinates     ▹*w*, *h*: width and height of bounding boxes     ▹x_max, y_max: bottom-right corner coordinates
**Read bounding boxes**  2: **for** each image and labels **do**  3:       Read bounding box (x_min,y_min,w,h) per labeled object in the image  4:       (x_max,y_max)←(x_min+w,y_min+h)  5:       Append to respective lists  6: **end for**
**Calculate ROI offsets**  7: $\left(x_{offset},y_{offset}\right)\leftarrow \left(\alpha \times \max\left(all\_w\right),\alpha \times \max\left(all\_h\right)\right)$
**Determine ROI corners then define source points**  8: $top\_left\leftarrow (\min\left(all\_x\_min\right)-x_{offset},\min\left(all\_y\_min\right)-y_{offset})$  9: $top\_right\leftarrow (\max\left(all\_x\_max\right)+x_{offset},\min\left(all\_y\_min\right)-y_{offset})$10: $bottom\_left\leftarrow (\min\left(all\_x\_min\right)-x_{offset},\max\left(all\_y\_max\right)+y_{offset})$11: $bottom\_right\leftarrow (\max\left(all\_x\_max\right)+x_{offset},\max\left(all\_y\_max\right)+y_{offset})$12: Source points src_pts←[top_left,top_right,bottom_left,bottom_right]
**Clip ROI corners to be within image boundaries** 13: **for** each point in src_pts **do** 14:       **if** $point_{x}<0$ **then** 15:          $point_{x}\leftarrow 0$ 16:       **else if** $point_{x}>image_{w}$ **then** 17:          $point_{x}\leftarrow image_{w}$18:       **end if**19:       **if** $point_{y}<0$ **then**20:          $point_{y}\leftarrow 0$21:       **else if** $point_{y}>image_{h}$ **then**22:          $point_{y}\leftarrow image_{h}$23:       **end if**24: **end for**
**Define target points based on image dimensions**25: Target points $trg\_pts\leftarrow [\left(0,0\right),\left(image_{w},0\right),\left(0,image_{h}\right),\left(image_{w},image_{h}\right)]$
**Calculate perspective transformation matrix**26: *M* = getPerspectiveTransform(src_pts, trg_pts)
**Transform images and bounding boxes**27: **for** each image and labels **do**28:       Transform image using *M*29:       Transform labels’ bounding box coordinates using *M*30:       Save the transformed images and bounding boxes31: **end for**
32: **return** *M*


Algorithm 1 automates the process of determining the ROI and generating the perspective transformation matrix *M*. The main steps of the algorithm are as follows:

1. **Initialize lists:** Store coordinates and dimensions of bounding boxes for all images, including minimum and maximum *x* and *y* coordinates, width, and height for each bounding box.
2. **Read bounding boxes:** Extract bounding box data from each image’s corresponding label file, calculate all ROI boundary points, and update the respective lists.
3. **Calculate offsets:** Determine the ROI offsets using a specific α value and the max width and height of all bounding boxes to define a slightly larger ROI.
4. **Determine ROI corners:** Use the minimum and maximum coordinates from the lists, along with the calculated offsets, to determine the corners of the ROI. These corners are the source points (src_pts) for the perspective transformation.
5. **Clip ROI corners:** Ensure ROI corners stay within image boundaries.
6. **Define target points:** Set target points (trg_pts) based on the image dimensions, representing the transformed image corners.
7. **Calculate transformation matrix:** Compute the perspective transformation matrix *M* using the source and target points. This matrix is used to transform the coordinates of the ROI to the new perspective.
8. **Transform images and bounding boxes:** Apply the transformation matrix *M* to each image and its bounding boxes. This involves transforming the image and adjusting the bounding box coordinates accordingly. The transformed images and bounding boxes are then saved.

The algorithm detailed in Algorithm 1 was initially applied to the training dataset to compute the perspective transformation matrix *M*. Subsequently, this matrix was utilized to transform the images and bounding boxes in the testing dataset. The proposed algorithm automates the perspective transformation process, ensuring consistent and precise selection of the region of interest (ROI) across extensive datasets. This automation minimizes manual intervention and the associated variability in selecting ROI corners for each image, making it an effective and scalable solution for applications like pothole detection in autonomous vehicles.

## 4. Experiment Design

### 4.1. Evaluation Dataset

**Figure 4:**
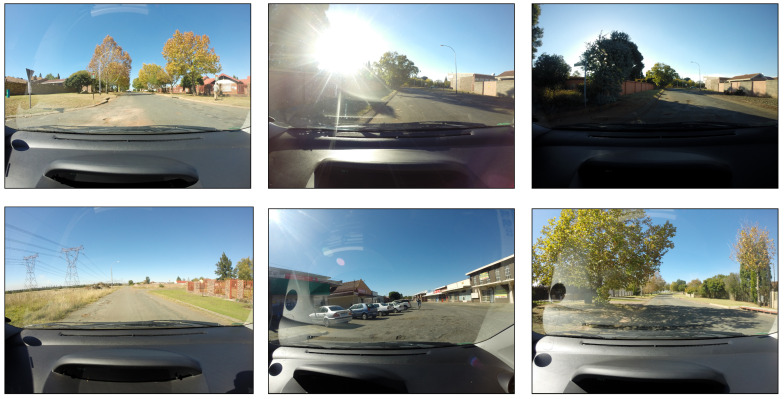
Demonstration of different samples of the dataset used in this work. These samples are some examples of the variance in lighting intensity and road conditions observed in this dataset.

To evaluate our proposed method, we utilized the dataset introduced by Nienaber et al. in [14,40]. This dataset, one of the few publicly available labeled pothole datasets, comprises 4405 images extracted from video footage captured with a GoPro camera mounted on a vehicle’s windshield. Unlike most of the other pothole datasets collected using mobile phones or drones, this dataset provides a realistic representation of South African road conditions from a driver’s perspective, making it particularly relevant for applications involving AVs and ground mobile robots. The dataset is split into two positive and negative directories. Positive samples are samples that include at least one instance of a pothole and comprise a total of 1119 images, while negative samples are samples without any potholes and comprise a total of 2658 images. Each image is provided with a label file with a bounding box’s format (class label, bounding box coordinates, width, and height). Moreover, the dataset is divided into training and testing subsets, with 628 images designated for testing. All images are provided in JPEG format with a resolution of 3680×2760 pixels. Figure 4 showcases six representative samples from the dataset, illustrating the challenges posed by varying illumination levels and pothole appearances, which are critical for developing robust and accurate pothole detection systems.

The dataset presents several significant challenges, particularly given the nature of the objects it aims to detect—potholes. Potholes vary widely in size, shape, and appearance, making them inherently difficult to detect. These variations are further exacerbated by the fact that potholes at greater distances appear smaller, complicating the task of accurately identifying them. Moreover, the color of potholes can differ depending on the surrounding environment, such as sandy areas, pavement, or other types of ground surfaces. This variation in appearance makes it challenging for a model to generalize across different scenarios, as the model must learn to recognize potholes in various contexts and lighting conditions. The difficulty of this task is amplified by the relatively small size of the dataset. Detecting small objects like potholes typically requires a large dataset to effectively learn the complex features necessary for accurate detection. To overcome this challenge, we use various augmentation techniques to enlarge the size of the training samples and enhance the robustness and accuracy of the pothole detection model. Furthermore, we used several augmentation techniques including affine scaling, rotation, and shearing to adjust the image size, orientation, and viewpoint to help the model recognize potholes of different sizes and angles. Horizontal flipping provides different perspectives, whereas Gaussian blur mimics motion blur to handle imperfect image captures. Adjustments to gamma contrast, brightness, and contrast normalization manage varying lighting conditions, ensuring that the model performs well under different environments. Additionally, additive Gaussian noise is added to make the model resilient to grainy images, and crop and pad transformations simulate occlusions and varying distances from the camera. As a result, the number of training images increased from 1119 images to 2658 images. These augmentations simulate real-world conditions, helping the model generalize better and improve pothole detection performance under diverse scenarios encountered by autonomous vehicles. By creating a diverse and representative training dataset, the model becomes more robust and capable of accurately detecting potholes in various challenging conditions.

### 4.2. Comparison Methods

Given the critical role of data quality and quantity in model performance, we explored several preprocessing methods to maximize the utility of the dataset in comparison to our proposed approach, auto transformation. The methods evaluated include “Image As Is”, ”Bottom Cropped”, and “Double Cropped”. The “Image As Is” method involves using the images without any alterations, while “Bottom Cropped” entails cropping the bottom portion of the images to exclude the dashboard, and “Double Cropped” involves cropping both the top and bottom parts of the images to remove the sky and dashboard. These preprocessing techniques were employed to eliminate extraneous elements, such as the dashboard and sky, which can lead to misclassifications and increased computational load. This step was crucial in optimizing the model’s efficiency and ensuring that the dataset provided the best possible training conditions for pothole detection.

To determine optimal cropping locations and minimize the loss of bounding boxes, we analyzed their distribution and found that 99% fell within the range of 1200–1800 pixels of their *y*-coordinates. Values above this range typically corresponded to the dashboard, while values below included sky regions.

**Figure 5:**
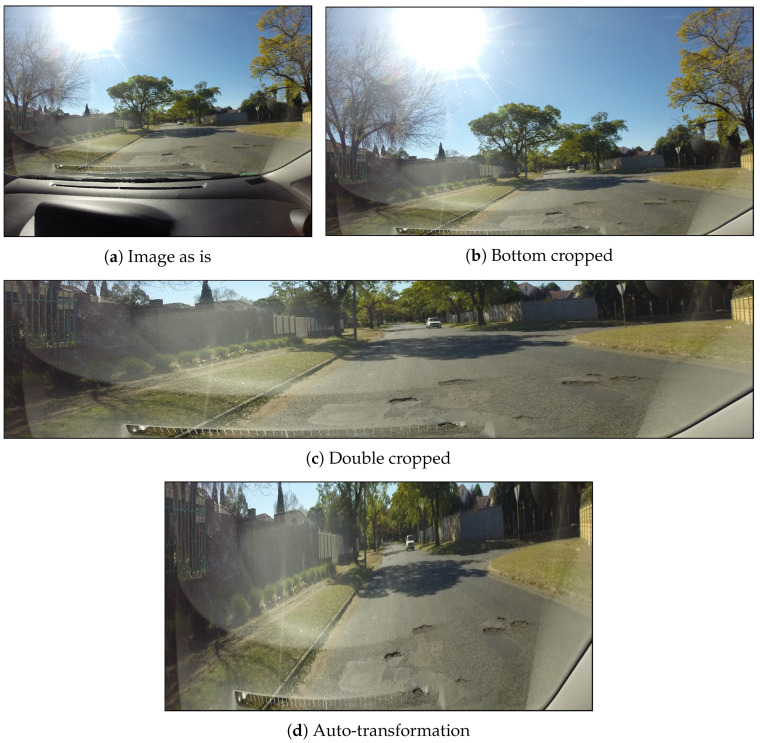
Visualization of the different comparison methods employed in our experiments: (**a**) Naive approach (image as is); (**b**) Fixed Cropping–Dashboard (bottom cropped); (**c**) Fixed Cropping–Dashboard and Sky Cropping (double-cropped); (**d**) Automated perspective transformation approach. The presented methods are demonstrated using the same input image.

Figure 5 visually compares the original image with two different cropping cases, in addition to the proposed method, demonstrating the impact of image composition on computational efficiency and model performance. This highlights the importance of preprocessing techniques in optimizing the detection pipeline. In Image As Is Figure 5a, the dashboard and sky occupy significant portions, introducing irrelevant information and increasing computational load. This results in unnecessary overhead and prolonged training times, negatively affecting model performance. To address this, Figure 5b, representing the Dashboard Cropping method, was cropped from the bottom to remove the dashboard, reducing false detections. However, this still left a substantial portion of the sky, contributing minimal information. Consequently, the Dashboard and Sky Cropping in Figure 5c was cropped to focus solely on the road surface, eliminating both the sky and the dashboard. Finally, Figure 5d shows our approach focusing only on the street where potholes are present.

### 4.3. Evaluation Metrics

In object detection, key evaluation metrics include intersection over union, precision, recall, average precision, and average recall. These metrics are crucial for assessing the performance of detection models.

**Intersection over union (IoU)** is a fundamental metric that measures the overlap between the predicted bounding box and the ground truth bounding box. It is calculated as the ratio of the area of intersection to the area of union of the two boxes, as shown in Equation (3). IoU is a threshold-based measure, typically used to determine whether a detection is considered a true positive (TP) or a false positive (FP).

(3) $$\mathrm{IoU}=\frac{\mathrm{Area}\;\mathrm{of}\;\mathrm{Intersection}}{\mathrm{Area}\;\mathrm{of}\;\mathrm{Union}}$$

**Precision** is the ratio of TPs to the sum of TPs and FPs, indicating the accuracy of the positive predictions made by the model. It is defined as shown in Equation (4).

(4) $$\mathrm{Precision}=\frac{TP}{TP+FP}$$

**Recall** measures the proportion of actual positives correctly identified by the model, calculated as the ratio of TPs to the sum of TPs and false negatives (FNs). This is expressed in Equation (5).

(5) $$\mathrm{Recall}=\frac{TP}{TP+FN}$$

**Average precision (AP)**, derived from the precision–recall curve, is calculated by integrating the area under this curve. AP at a specific IoU threshold (e.g., AP_50_ for IoU ≥ 0.5) represents the precision averaged across different recall levels at that threshold. AP_50:95_ refers to the average precision computed at multiple IoU thresholds ranging from 0.5 to 0.95 with a step size of 0.05. This metric provides a comprehensive evaluation of model performance across various IoU thresholds. AP_50_ and AP_75_ specifically denote AP at IoU thresholds of 0.5 and 0.75, respectively, offering insights into model precision at different levels of overlap criteria.

**Average recall (AR)** reflects the average recall over different numbers of detections per image, providing an aggregate measure of the model’s ability to identify relevant instances among all actual positives. AR_max=1_ and AR_max=10_ denote the average recall when considering a maximum of one detection per image and ten detections per image, respectively, across IoU thresholds of 0.5 to 0.95. These metrics help to evaluate the model’s recall capability, considering different levels of detection strictness.

These metrics collectively offer a detailed assessment of the detection model’s performance, highlighting its strengths and weaknesses across various detection thresholds and conditions.

### 4.4. Evaluation Strategy

To assess the effectiveness of our proposed method for detecting potholes at varying distances, we employed two evaluation strategies. Initially, we evaluated the performance using a single class (pothole). Subsequently, we expanded the analysis to include three classes (near, medium, and far) by categorizing the bounding boxes based on the *y*-coordinates of their top-left corners, with each region representing a different class. This classification aimed to measure the effectiveness of our approach in enhancing the detection of potholes at different distances. We conducted a comparative analysis against other dataset processing techniques, applying these evaluation strategies to each dataset using predefined thresholds as follows:

For the Image As Is and the cropping methods:

- **Far:** y≤1350
- **Medium:** 1351≤y≤1500
- **Near:** 1501≤y

For the Automatic Transformation approach:

- **Far:** y≤670
- **Medium:** 671≤y≤1099
- **Near:** 1100≤y

### 4.5. Implementation Settings

Throughout all of our experiments, we trained the object detection models using the following hyperparameters: 100 epochs, a stochastic gradient descent (SGD) optimizer, a batch size of 16, and a learning rate of 0.01. The learning rate determines the step size, which is the amount the model’s parameters are adjusted with respect to the gradient during optimization. This rate was chosen to balance the speed of convergence with stability, ensuring the step size is neither too large, causing overshooting; nor too small, leading to slow convergence. The SGD optimizer was selected based on its superior performance compared to the ADAM optimizer in our tests. Additionally, after data augmentation, the dataset, consisting of 2658 images, was divided into 80% for training and 20% for validation. For the experiments, we utilized the small, medium, and large variants of YOLOv5 to assess the model’s performance across different scales and complexities. The best-performing model on the validation set, determined based on the results from each epoch, was selected as the final model. During the evaluation, we used a confidence threshold of 0.5 to filter out low-confidence detections and a non-maximum suppression (NMS) threshold of 0.45 to eliminate redundant overlapping detections. These thresholds were chosen to optimize the balance between precision and recall, contributing to the robustness of the final detection results.

The hardware setup for our experiments consisted of a GTX 1080 GPU with 11 GB of memory, an Intel i7-8700K CPU, and 64 GB of RAM. All experiments utilized images that were downscaled to a resolution of 800×800 pixels, from the original 3680×2760 resolution captured by a high-resolution camera, while maintaining their aspect ratio [28]. We chose an input resolution of 800×800, which is marginally larger than the default YOLOv5 base resolution of 640×640. This choice was justified based on our empirical findings, where the larger input size allowed for better feature representation, especially for detecting smaller and more distant potholes. The increased resolution facilitated the model’s ability to capture finer details, thus enhancing detection accuracy without significantly compromising computational efficiency or overwhelming the available hardware resources.

To start the training process, we fine-tuned the YOLOv5 model on our dataset, leveraging the pre-trained weights and further training them specifically on our data. The default training augmentations provided by the YOLOv5 framework were employed throughout the experiments, alongside the default hyperparameters, which we kept unchanged for consistency and standardization purposes except for the ones we mentioned earlier. The training process exclusively utilized positive images, applying the preprocessing augmentations as detailed in Section 4.1. This methodological choice, including the exclusion of negative images, is further substantiated by an ablation study presented in the following section, which validates the effectiveness of these decisions in optimizing the model’s performance. Additionally, based on our experiments and achieving optimal transformation outcomes, we selected an α value of 0.2 for the automatic transformation algorithm presented in Algorithm 1.

## 5. Results and Discussion

### 5.1. Experiment 1: Naive vs. Fixed Cropping vs. Automated Transformation Approach

In this experiment, we systematically trained one variant of YOLOv5 (small) on all possible dataset configurations. These configurations were evaluated for both single-class (pothole) and multi-class (near, medium, and far distance) detection tasks. As detailed in Table 1. Each method was assessed for overall pothole detection as one class, and for each distance-based class separately.

As illustrated in Table 1, our novel approach demonstrates superior performance across all metrics, surpassing all other methods. Notably, we observed a substantial increase in AP_50:95_, with a 43% improvement in the single class using our proposed approach compared to the Image As Is method. Furthermore, there were increases of 34%, 63%, and 194% for the near, medium, and far classes, respectively, at the same IoU threshold. Additionally, our approach resulted in an improvement in AP_50_ of 30%, and in AP_75_ of 73%. Improvements were also observed in AR, with AR_*max*=1_ increasing by 26% and AR_*max*=10_ by 36%. These results underscore the effectiveness of our method in enhancing pothole detection accuracy compared to traditional approaches.

The significant improvements observed in our results are due to the effectiveness of the automatic perspective transformation approach, which virtually brings potholes closer to the vehicle, amplifying their features and making them more discernible to the detection model, as Figure 3 shows. This perspective adjustment enhances the model’s ability to learn and recognize pothole patterns, resulting in more accurate detections. The amplification of pothole features simplifies the learning process for the YOLOv5 model, leading to significant improvements in average precision across various classes and IoU thresholds.

The proposed approach not only improved the overall pothole detection performance but also excelled in detecting the more challenging cases, particularly medium- and far-distance potholes, which are the most critical for safety. Our method significantly improved the detection accuracy for far potholes, an area where other methods have notably underperformed. The consistent performance gains across different IoU thresholds validate the robustness of our approach. Traditional detection methods struggle with varying perspectives and angles, while our method standardizes these perspectives, offering a more uniform dataset for the model to train on, which is crucial for real-world applications. The success of our approach in enhancing pothole detection accuracy has broader implications for other object detection tasks, potentially leading to advancements in multiple areas of computer vision.

### 5.2. Experiment 2: Effects of Network Complexity/Scale on Performance

As demonstrated in Table 2, we performed an extensive evaluation of our proposed approach compared to the baseline method, where the image remains unchanged. The evaluation was performed using three variants of YOLOv5 (small, medium, and large). Furthermore, we tested these models on three distinct classes (near, medium, and far), as well as on the entire pothole dataset treated as a single class (pothole).

The results demonstrate a significant improvement in detection accuracy when comparing the two approaches across all YOLOv5 variants for all classes. Notably, the YOLOv5-small variant, when applied to our approach, outperformed the YOLOv5-large variant applied to the baseline approach, knowing that YOLOv5-large has almost six times the number of parameters of YOLOv5-small, as explained in Section 3.1.2. This highlights the effectiveness of our method in detecting potholes across various distances while requiring lower computational resources compared to the traditional approach.

The results in Table 2 reveal that our proposed approach significantly enhances detection accuracy across all YOLOv5 variants for every class compared to the baseline method. The better performance of the YOLOv5-small variant under the AP_50_ metric is particularly noteworthy, which outperformed both the medium and large variants when using our method. We hypothesize that this counterintuitive result stems from the larger and medium YOLOv5 models being more susceptible to the poor quality of some labels, potentially learning and incorporating these inaccuracies into their detection processes more than the smaller variant under the lower IoU threshold. Consequently, the small version’s relatively simpler architecture may have enabled it to generalize better and avoid overfitting to the noisy data, resulting in enhanced detection accuracy [41]. This finding underscores the effectiveness of our automatic perspective transformation approach and suggests that smaller, less complex models can be more robust in scenarios where data quality is variable, offering valuable insights for similar projects in object detection.

### 5.3. Experiment 3: Ablation Study

Given that our dataset contains negative images and the augmentation capabilities inherent in the YOLOv5 framework, we conducted a comprehensive series of experiments to identify the optimal configuration for training our model. The goal was to quantitatively validate the chosen configuration throughout our experiments. We integrated the negative images with the positive images and explored various augmentation strategies, testing the effectiveness of relying exclusively on the YOLOv5 framework’s default augmentations versus supplementing them with additional manual augmentations that were introduced in Section 4.1. This methodological investigation aimed to rigorously assess the impact of these different approaches on the model’s performance.

To identify the optimal configuration, we created and evaluated four distinct setups using our automatic perspective transformation approach. The results of these experiments are detailed in Table 3. The chosen configuration was then utilized for subsequent experiments presented in Table 1 and Table 2.

The four configurations tested were as follows:

1. YOLOv5’s augmentations only without negative images: This setup utilized only YOLOv5’s augmentation step without negative images, as illustrated in the first row of Table 3.
2. YOLOv5’s augmentations with negative images: This setup included negative images alongside YOLOv5’s augmentation step, shown in the second row of Table 3.
3. Manual preprocessing and YOLOv5’s augmentations without negative images: This configuration combined manual preprocessing augmentations with YOLOv5’s augmentations, using only positive images. It achieved the best results among all setups.
4. Manual preprocessing and YOLOv5’s augmentation with negative images: This setup used both manual and YOLOv5’s augmentations, incorporating negative images into the positive dataset. It resulted in the lowest performance metrics.

As illustrated in Table 3, the configuration that utilizes manual pre-processing with only the positive dataset during training consistently achieved the best results in all metrics. This approach was subsequently applied to all experiments conducted in this study, confirming its superiority as the optimal method for improving the accuracy of pothole detection.

Moreover, the results show that our approach that combined manual preprocessing with only the positive dataset consistently outperformed all other configurations to improve the accuracy of the pothole detection. This setup, which excluded negative images and relied on extensive augmentations, proved superior across all metrics. We hypothesize that the inclusion of negative images introduced noise into the training process, as these images lack bounding boxes or pothole features, which are critical for improving the model’s robustness and pattern recognition capabilities. Additionally, the extensive use of manual preprocessing augmentations exposed the model to a wider variety of pothole shapes, colors, and orientations, enhancing its ability to generalize across different scenarios. In contrast, relying solely on YOLOv5’s framework augmentation step limited the model’s exposure to diverse cases, thereby restricting its generalization potential.

### 5.4. Computational Latency Analysis

To validate the real-world feasibility of our approach, we perform a computational latency analysis of the main components in our pothole detection pipeline. We measure the average latency of the fine-tuned models’ inference times during evaluation, as well as the latencies of pre-processing, perspective transformation, and post-processing stages. The pre-processing stage primarily comprises image normalization and rescaling processes, while the post-processing step includes filtering the resulting detections using NMS. For model inference times, we evaluate the three fine-tuned YOLOv5 variants (i.e., small, medium, large) produced by our auto-transformation approach, as presented in Table 2. Notably, the inference times of the other methods should be the same, given that the generated model architectures are identical except for their training weights.

For model inference times, we evaluate the three fine-tuned YOLOv5 variants (i.e., small, medium, large) produced by our auto-transformation approach, as presented in Table 2. Notably, the inference times of the other methods should be the same, given that the generated model architectures are identical except for their training weights.

As demonstrated in Table 4, our proposed perspective transformation approach adds a small computational latency overhead relative to the inference times of the object detection models, with an average processing time of 14.4 ms. This latency arises primarily because we directly apply the perspective transformation on the raw high-resolution images (3860×2760 pixels) using bilinear interpolation. Through further experimentation, we observed that the perspective transformation time is directly proportional to the input image resolution. Notably, this latency can be reduced to 4 ms by downscaling the image by half across each dimension (i.e., height/2 and width/2) before applying the transformation, which should have a negligible effect on the object detection performance due to this additional rescaling step. We recommend that the developers adjust the input resolution based on their timing requirements. Finally, we note that our implementation utilized OpenCV’s warpPerspective() function in Python 3.9. Using a C++ implementation of this function can significantly improve performance and minimize the resulting latency. However, we leave further optimization to future work.

Despite the small overhead, this technique significantly enhances pothole detection performance, as evidenced by our experimental results. This demonstrates the efficiency and effectiveness of incorporating perspective transformation in our detection pipeline.

## 6. Conclusions

In this paper, we introduced a novel method for improving pothole detection by leveraging perspective transformation to automatically extract ROI from images and their corresponding labels. The transformed dataset was then fed into the YOLOv5-small object detection model. Our approach resulted in a notable improvement in detection accuracy using YOLOv5-small, achieving a 43% increase in AP for a single class at IoU thresholds of 0.5 to 0.95 (AP_50:95_), compared to the naive use of unchanged images. Similarly, improvements of 29% and 32% in the same metric for YOLOv5-medium and YOLOv5-large have been achieved, respectively. In addition, the method significantly improved the detection of potholes at various distances, addressing a crucial aspect of road safety, where it has achieved significant increases of 34%, 63%, and 194% in the same metric (AP_50:95_) for near, medium, and far, respectively. Moreover, Table 2 shows further improvement using both YOLOv5-medium and YOLOv5-large. The findings underscore the critical role of preprocessing techniques, such as perspective transformation, in enhancing the performance of object detection tasks.

For future work, we propose developing a deep-learning model capable of dynamically regressing the four corner points of the street in each image to generate a perspective transformation matrix. This approach would necessitate labeled data, potentially obtainable from semantic segmentation datasets, to further automate and refine the pre-processing pipeline.

## Figures and Tables

**Table 1:** Experiment 1 results. This experiment compares the different approaches presented in this work by fine-tuning YOLOv5-small under each configuration and then evaluating their performance on the test set using various object detection metrics. Our proposed approach demonstrates superior performance across all metrics and pothole distance categories.

Approach	Pothole Distance	Metric (%)				
		**AP_50:95_**	**AP_50_**	**AP_75_**	**AR_*max*=1_**	**AR_*max*=10_**
Image as Is	All	19.8	47.7	12.8	17.1	25.8
	Near	23.6	55.0	15.1	20.7	29.9
	Medium	19.5	48.4	13.5	21.6	26.6
	Far	6.8	18.8	3.1	10.0	11.5
Bottom Cropped	All	18.2	46.1	10.2	15.9	23.7
	Near	21.4	51.7	12.2	19.3	27.3
	Medium	18.0	46.1	11.1	20.6	25.4
	Far	5.5	18.2	1.5	7.3	9.0
Double Cropped	All	17.5	46.8	9.3	15.6	23.0
	Near	21.7	58.6	11.0	19.4	27.6
	Medium	17.5	47.9	10.5	20.0	23.9
	Far	11.2	27.9	5.1	11.7	14.2
Auto Transformation	All	**28.4**	**61.9**	**22.1**	**21.6**	**35.2**
	Near	**31.7**	**64.5**	**26.5**	**26.2**	**38.5**
	Medium	**31.7**	**68.8**	**26.0**	**31.9**	**38.8**
	Far	**20.0**	**50.0**	**12.0**	**18.8**	**27.4**

The best result per metric and pothole distance category is highlighted in **bold**.

**Table 2:** Experiment 2 results. This experiment compares the naive (i.e., Image As Is) approach with our proposed approach on three YOLOv5 variants. In each configuration, a YOLOv5 variant is fine-tuned on the corresponding approach’s training set and then evaluated on the test set using various object detection metrics. Results show that our proposed approach always surpasses the performance of the naive approach regardless of the utilized variant. Additionally, combining YOLOv5-small with our proposed approach significantly outperforms the naive approach even when compared to the YOLOv5-large configuration, for which the model is over six times larger in terms of the number of parameters, across all metrics and pothole distance categories.

Approach	Object Detection Model	Parameters (M)	FLOPs (G)	Pothole Distance	Metric (%)				
					**AP_50:95_**	**AP_50_**	**AP_75_**	**AR_*max*=1_**	**AR_*max*=10_**
Image As Is	YOLOv5-small	7.2	16.5	All	19.8	47.7	12.8	17.1	25.8
				Near	23.6	55.0	15.1	20.7	29.9
				Medium	19.5	48.4	13.5	21.6	26.6
				Far	6.8	18.8	3.1	10.0	11.5
	YOLOv5-medium	21.2	49.0	All	21.7	50.3	14.6	17.7	27.4
				Near	25.0	55.1	18.1	21.1	31.1
				Medium	21.9	51.8	15.1	22.9	29.3
				Far	7.4	25.0	1.7	9.6	12.3
	YOLOv5-large	46.5	109.1	All	21.7	50.5	14.2	17.5	27.7
				Near	25.4	55.7	18.4	21.2	31.6
				Medium	21.6	54.9	11.6	22.9	29.9
				Far	6.7	20.5	2.8	9.8	12.0
Auto Transformation	YOLOv5-small	7.2	16.5	All	28.4	**61.9**	22.1	21.6	35.2
				Near	31.7	**64.5**	26.5	**26.2**	38.5
				Medium	**31.7**	**68.8**	26.0	**31.9**	**38.8**
				Far	20.0	**50.0**	12.0	18.8	27.4
	YOLOv5-medium	21.2	49.0	All	28.0	61.6	22.4	21.5	34.5
				Near	31.1	62.9	28.3	25.9	**38.6**
				Medium	30.7	67.9	24.5	30.1	37.6
				Far	19.3	47.9	12.2	18.8	25.9
	YOLOv5-large	46.5	109.1	All	**28.6**	60.2	**24.5**	**22.0**	**35.3**
				Near	**32.0**	63.8	**29.6**	25.9	38.5
				Medium	30.9	65.0	**27.7**	29.7	37.3
				Far	**20.8**	47.6	**13.8**	**21.0**	**29.1**

The best result per metric and pothole distance is highlighted in **bold**.

**Table 3:** Ablation study results comparing the different preprocessing configurations. These results are based on the automated transformation approach on all test set potholes using YOLOv5-small. Including the preprocessing augmentations while excluding the negative samples (training images without potholes) produced the best performance across all metrics.

Configuration		Metric (%)				
**Preproc. Augs.**	**Neg. Images**	**AP_50:95_**	**AP_50_**	**AP_75_**	**AR_*max*=1_**	**AR_*max*=10_**
		27.1	59.7	20.1	19.6	34.6
	✓	26.2	57.3	18.9	20.5	32.1
✓		**28.4**	**61.9**	**22.1**	**21.6**	**35.2**
✓	✓	25.8	55.1	19.0	19.8	31.3

The best result per metric is highlighted in **bold**.

**Table 4:** Computational latency analysis of different components within the pothole detection pipeline across various YOLOv5 model variants. Specifically, we measure the average latency of each of the pre-processing, perspective transformation, inference, and post-processing stages.

Object Detection Model	Pre-Processing (ms)	Perspective Transformation (ms)	Inference (ms)	Post-Processing (ms)	Total Time (ms)
YOLOv5-small	0.6	14.4	**7.2**	**1.2**	**23.4**
YOLOv5-medium	0.6	14.4	14.3	1.4	30.7
YOLOv5-large	0.6	14.4	25.5	1.3	41.8

The best result is highlighted in **bold**.

## Data Availability

The original data presented in the study are openly available in Kaggle at https://www.kaggle.com/datasets/sovitrath/road-pothole-images-for-pothole-detection/data (accessed on 1 August 2024).
